# Supplementary material for: Bacterial sensitivity to chlorhexidine and povidone-iodine antiseptics over time: a systematic review and meta-analysis of human-derived data
Source: Sci Rep. 2023 Jan 7;13:347. doi: 10.1038/s41598-022-26658-1 (PMC9825506; doi:10.1038/s41598-022-26658-1)
Supplement: Supplementary file 1 — Supplementary Information 1. [file 41598_2022_26658_MOESM1_ESM.pdf]

Appendix 1. Search Strategy

```
# https://github.com/ropensci/medrxivr

install.packages("medrxivr") devtools::install_github("ropensci/medrxivr")

library(medrxivr)
setwd ("/Users/ryckiewade/OneDrive - University of Leeds/Chlorhex resistance/SR/searches")
options(scipen = 999) #show large numbers, suppress the e+

###########
# medRxiv #
###########

# Option a) Get a copy of the database from the live medRxiv API endpoint; more up-to-date but less
quick/reliable
preprint_data <- mx_api_content()

# Option b) Get a copy of the database from the daily snapshot; more reliable but only updated daily
preprint_data <- mx_snapshot()

###########
# bioRxiv #
###########

# Get a copy of the database from the live bioRxiv API endpoint preprint_data <-
mx_api_content(server = "biorxiv")

############
# Searches #
############

# Perform a simple search
results <- mx_search(data = preprint_data,

                    query ="dementia")

# Perform an advanced search
topic1 <- c("chx", "chlorhex*", "chloraprep", "iodin*", "iodophor", "betadine", "betadine", "povidone")
# Combined with Boolean OR
topic2 <- c("resistan*", "MIC", "minimum inhibitory")
Boolean OR
myquery <- list(topic1, topic2)
# Combined with Boolean AND

results <- mx_search(data = preprint_data, query = myquery)

# Export to .BIB file mx_export(data = results,

file = "mx_search_results.bib")

# Download PDFs for records returned by search mx_download(results, # Object returned by
mx_search(), above

# Combined with
```

"pdf_preprints/", # Directory to save PDFs to create = TRUE) # Create the directory if it doesn't exist
