# Supplementary material for: Bacterial sensitivity to chlorhexidine and povidone-iodine antiseptics over time: a systematic review and meta-analysis of human-derived data
Source: Sci Rep. 2023 Jan 7;13:347. doi: 10.1038/s41598-022-26658-1 (PMC9825506; doi:10.1038/s41598-022-26658-1)

Table S1. Summary of study characteristics

	MSSA	MRSA	Other Staph	Strep	Enterobacteriales	Anaerobes	Enterococci	Pseudomonas	Other	All
**Studies**	27	29	27	69	58	24	43	21	54	352
**Microbes**	3460	522	352	153	965	77	412	109	168	6218
**Country**	1 was global						1 global			2 Global
N. America	4 (15%)	4 (14%)	3 (11%)	19 (28%)	8 (14%)	8 (33%)	8 (19%)	3 (14%)	13 (24%)	70 (20%)
S. America				18 (26%)		8 (33%)	5 (12%)	1 (5%)	14 (26%)	46 (13%)
Europe	17 (63%)	24 (83%)	20 (74%)	9 (13%)	31 (53%)	4 (17%)	17 (40%)	14 (67%)	15 (28%)	151 (43%)
Asia	5 (19%)	1 (3%)	4 (15%)	23 (33%)	19 (33%)	4 (17%)	12 (28%)	3 (14%)	12(22%)	83 (24%)
**Year**										
1950-1970	1 (4%)		1 (4%)							2 (0.6%)
1971-1980	1 (4%)				6 (10%)			2 (10%)		9 (2.6%)
1981-1990		1 (3%)								1 (0.3%)
1991-2000		4 (14%)		2 (3%)						6 (1.7%)
2001-2005	2 (7%)	3 (10%)	1 (4%)	4 (6%)	3 (5%)	6 (25%)	1 (2%)	1 (5%)	7 (13%)	28 (8%)
2005-2010	5 (19%)	7 (24%)	3 (11%)	1 (1%)	9 (16%)	1 (4%)	11 (26%)	6 (29%)	9 (17%)	52 (15%)
2011-2015	13 (48%)	10 (34%)	18 (67%)	15 (22%)	20 (34%)	1 (4%)	8 (19%)	6 (29%)	10 (19%)	101 (29%)
2016-2017	1 (4%)	1 (3%)		22 (32%)	1 (2%)	12 (50%)	12 (28%)	2 (10%)	17 (31%)	68 (19%)
2018-2019	3 (11%)	3 (10%)	4 (15%)	19 (28%)	7 (12%)	2 (8%)	6 (14%)	2 (10%)	8 (15%)	54 (15%)
2020-2021	1 (4%)			6 (9%)	12 (21%)	2 (8%)	5 (12%)	2 (10%)	3 (6%)	31 (9%)
**PIV/CLHX**	2/25	1/28	1/26	0/69	13/45	1/23	4/39	3/18	1/53	26/326 7%/%93
**Isolate nature (lab/clinical)**	16/11	5/24	9/18	55/14	26/32	18/6	29/14	13/8	43/11	214/138 61%/39%
**MDR**	0	3 (10.3%)	0	0	6 (10%)	0	3 (7%)	2 (10%)	2 (4%)	16 (5%)
**Mean Exposure Time**	2066 (1261.53)	2312 (692.00)	3007 (1842.46)	1596 (598.53)	1980 (1213.90)	3243 (1721.82)	1913 (1051.75)	2080 (1376.37)	1973 (804.95)	2100 (1224.18)

Table S2. The number of experiments captured from the included studies, tabulated by the antiseptic studied and the family of the microbe

Family	Number of experiments	
	Chlorhexidine	Povidone iodine
Acinetobacter	6	0
Actinomyces	1	0
Anaerobes	10	1
Enterococci	19	2
Enteriobacteriales	24	13
Pseudomonas	12	3
Pyogenes (Group A, β-haemolytic) Streptococci	3	0
Viridans (Group B, α-haemolytic) Streptococci	22	0
Coagulase Negative Staphylococci	15	1
Staphylococcus aureus (unspecified)	3	0
Staphylococcus aureus (MRSA)	23	1
Staphylococcus aureus (MSSA)	13	2

Figure S1. Forest plot of the mean MBC for chlorhexidine, in different *Staphylococci*

studyid	ES (95% CI)	% Weight
Staphylococcus Aureus (MSSA)		
Antonelli 2019	2 (1, 2)	23.98
Cowley 2015	5 (1, 9)	14.70
Forbes 2014	13 (5, 21)	6.73
Furi 2013	5 (5, 5)	24.22
Hardy 2018	6 (2, 9)	15.68
Touzel 2016	1 (-17, 19)	1.58
Xing 2011	8 (-5, 21)	2.80
Xing 2011	19 (13, 24)	10.32
Subtotal (I-squared = 95.3%, p = 0.000)	6 (4, 9)	100.00
.		
Staphylococcus Aureus (MRSA)		
Conceicao 2015	1 (1, 1)	9.33
Conceicao 2015	1 (1, 2)	8.77
Conceicao 2015	1 (1, 1)	9.59
Conceicao 2015	1 (1, 2)	6.64
Conceicao 2015	1 (1, 1)	9.51
Conceicao 2015	1 (1, 2)	8.02
Conceicao 2015	1 (1, 1)	9.54
Conceicao 2015	2 (1, 2)	8.63
Conceicao 2015	1 (1, 1)	9.49
Conceicao 2015	1 (1, 1)	9.59
Conceicao 2015	2 (1, 2)	7.05
Hardy 2018	9 (6, 11)	1.63
Hardy 2018	16 (14, 19)	1.22
Hardy 2018	16 (12, 19)	0.75
McGann 2011	35 (21, 50)	0.06
Tetz 2015	4 (-4, 12)	0.18
Subtotal (I-squared = 94.0%, p = 0.000)	2 (1, 2)	100.00
.		
Coagulase Negative Staphylococci		
Forbes 2014	2 (1, 2)	38.91
Forbes 2014	4 (2, 6)	33.28
Forbes 2014	34 (10, 57)	1.40
Skovgaard 2013	6 (2, 9)	23.76
Tambe 2001	2 (-15, 19)	2.65
Subtotal (I-squared = 75.9%, p = 0.002)	4 (1, 7)	100.00

NOTE: Weights are from random effects analysis

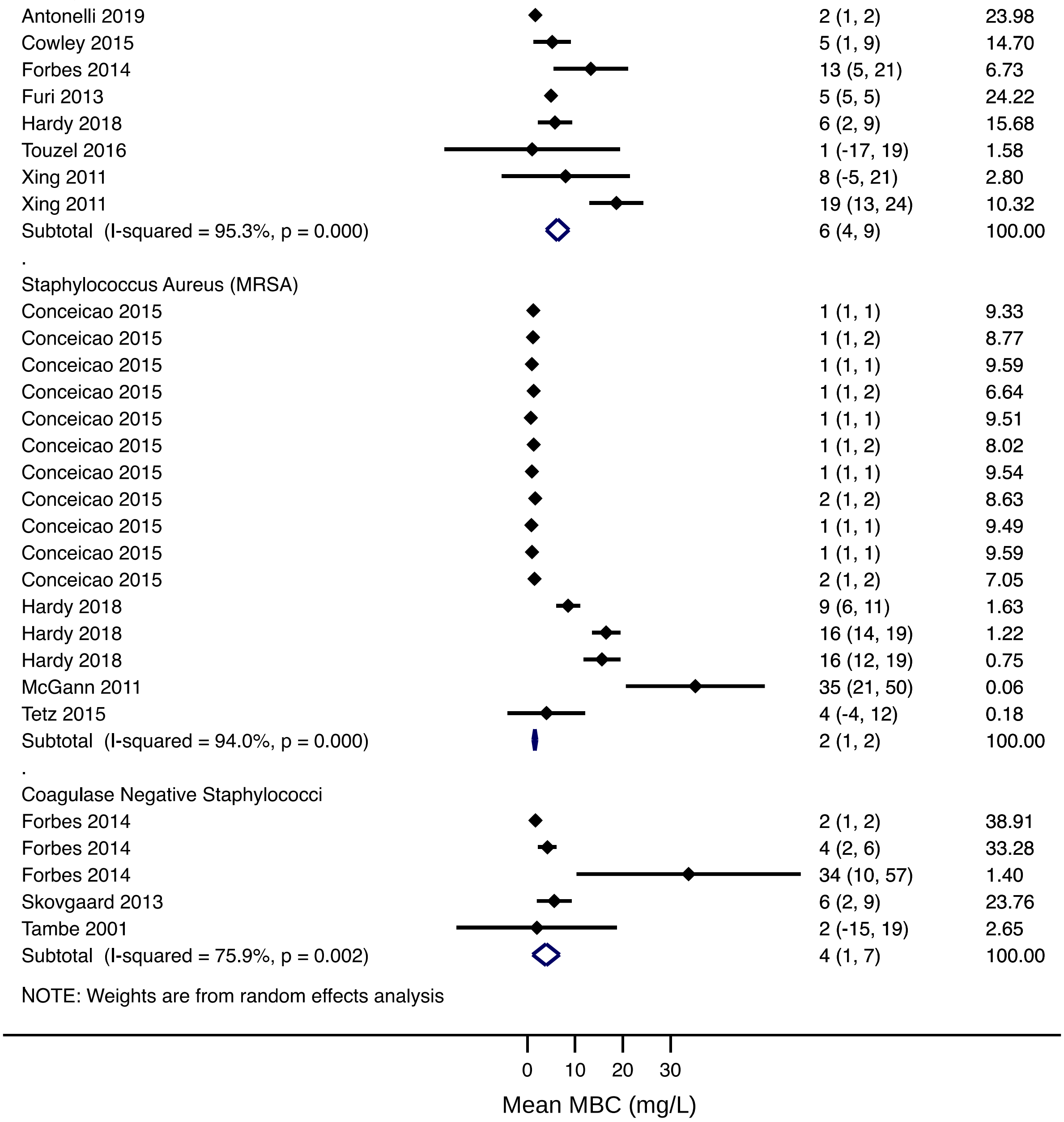

Supplement: Supplementary file 2 — Supplementary Information 2. [file 41598_2022_26658_MOESM2_ESM.pdf]
